# A Case of Primary Ciliary Dyskinesia Caused by *CFTR* Gene and *SFTPC* Gene Variants

**DOI:** 10.1155/carm/2903077

**Published:** 2026-08-24

**Authors:** Xinhui Yuan, Dan Shao, Yumei Li, Jizu Ling, Yigu Gong

**Affiliations:** ^1^ Pediatrics, Lanzhou University First Hospital, Lanzhou 730000, Gansu, China, lzu.edu.cn; ^2^ Department of Child Health, First Hospital of Lanzhou University, Lanzhou 73000, Gansu, China, lzu.edu.cn

**Keywords:** children, genetics, primary ciliary dyskinesia

## Abstract

**Introduction:**

This study retrospectively analyzed the clinical data of a female child with primary ciliary dyskinesia (PCD) caused by variants in the CFTR (NM_000492.3) and SFTPC (NM_003018.3). All variant descriptions in this report follow the HGVS standardized nomenclature guidelines. These two variants are considered to be potentially associated with pediatric PCD.

**Case Presentation:**

A 13‐year‐old Chinese female patient with a history of recurrent respiratory infections was admitted due to intermittent cough and sputum production for one month, exacerbated by fever for five days. Chest CT revealed a lung infection and bronchiectasis. Transmission electron microscopy of ciliary biopsy identified structural abnormalities in bronchial mucosa cilia, including aberrant microtubule arrangements (8 + 2, 9 + 1, and 7 + 2 patterns), and disorganized peripheral microtubules. The whole‐exome sequencing (WES) identified a heterozygous variant c.374T > C (p.Ile125Thr) in CFTR (NM_000492.3) and a heterozygous variant c.115G > T (p.Val39Leu) in SFTPC (NM_003018.3), with all nomenclature complying with HGVS recommendations. A comprehensive diagnosis of PCD was confirmed. The patient improved after anti‐infective and symptomatic therapy and was discharged.

**Conclusion:**

Variants in the CFTR and SFTPC genes may be associated with PCD in children. This case highlights the importance of early genetic variant testing and ciliary ultrastructural analysis (e.g., transmission electron microscopy) in children with recurrent respiratory tract infections, bronchiectasis, or chronic sinusitis, thereby facilitating timely diagnosis and clinical intervention.

## 1. Introduction

Primary ciliary dyskinesia (PCD) is a rare genetic ciliary structural defect disease. Its genetic model is mainly autosomal recessive inheritance, and X‐linked recessive inheritance and autosomal dominant inheritance have occasionally been reported [1]. The main pathogenesis is the variation of genes encoding cilia components, causing changes in the typical structure of the cilia, which leads to dysfunction of the cilia and may cause dysfunction of the tissues or organs related to cilia in the human body. Clinically, patients may manifest repeated respiratory infections and bronchodilation, while some can experience visceral translocation, otitis media, infertility, or hydrocephalus [2]. We report a case of PCD caused by CFTR (c.374T > C, NM_000492.3) and SFTPC (c.115G > T, NM_003018.3) gene variants and review‐related literature to enhance clinicians’ recognition of genotype–phenotype correlations in this disease.

## 2. Case Presentation

**FIGURE 1:**
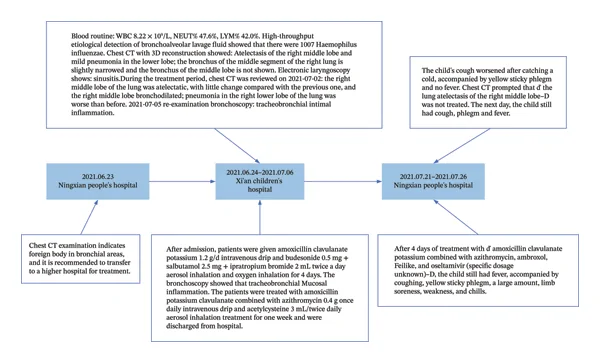
Time axis of disease course.

The child, a female 13 years old, was admitted to the pediatrics department of our hospital because of “intermittent cough and expectoration for 1 month, aggravation with fever for 5 days.” One month prior to admission, the patient developed paroxysmal cough and six episodes of hemoptysis without an apparent cause. Associated symptoms included small‐volume dark‐red mucus expectoration, fatigue, poor spirit, night sweats, sore throat, hoarseness, nausea, vomiting, abdominal distension and abdominal pain. She had no fever, chills, headache or dizziness. The detailed diagnostic and therapeutic process is presented in Figure 1. Before admission, there was still fever, accompanied by cough, yellow sticky sputum, a larger amount, aching limbs, weakness, and chills. During the disease, the child was mentally conscious, had a poor diet, had good sleep at night, had a dry stool for 1‐2 days/time, and had normal urination.

### 2.1. Birth and Developmental History

The patient is G3P3 (gravida 3, para 3), born full‐term via spontaneous vaginal delivery, and exclusively breastfed postpartum. The parents are nonconsanguineous with no family history of genetic disorders or consanguinity. The child’s growth and development have been consistent with age‐appropriate milestones.

### 2.2. Physical Examination on Admission

Temperature is 38°C, heart rate is 86 beats/min, respiratory rate is 23 breaths/min, blood pressure is 114/66 mmHg, and weight is 39.9 kg. The patient was conscious and in stable condition with smooth respiration. Conjunctival congestion was observed, with no tonsillar enlargement. Bilateral respiratory movements were symmetrical, and no chest retractions were noted. Auscultation revealed coarse breath sounds in the left lung and decreased breath sounds in the right middle lobe, with no significant dry or moist rales detected.

### 2.3. Auxiliary Examinations

Routine blood, urine, and stool tests were all normal. CRP is 5.75 mg/L. Liver function: Transaminases were normal, alkaline phosphatase is 190 U/L, and total bile acid is 28.6 μmol/L. Electrolytes are K 3.33 mmol/L, anion gap is 20.2 mmol/L, erythrocyte sedimentation rate is 46 mm/h, and procalcitonin is 0.05 ng/mL. Respiratory pathogen nucleic acid microarray testing yielded positive results for Mycoplasma pneumoniae DNA. Abdominal ultrasound showed normal findings. Abdominal lymph node ultrasound revealed multiple lymph nodes within the abdominal cavity and bilateral inguinal regions. Neck and supraclavicular lymph node ultrasound demonstrated multiple enlarged lymph nodes in the bilateral cervical areas. Gynecological ultrasound detected no significant abnormalities of the uterus or bilateral adnexa. Pulmonary function tests showed reduced FEV1 and vital capacity, suggesting restrictive ventilatory dysfunction. The FEV1/FVC ratio was normal, with no evidence of obstructive factors; low peak flow indicated possible significant airway obstruction, together with reduced end‐expiratory parameters and small‐airway dysfunction. The severity was classified as moderate pulmonary ventilation impairment. Cardiac ultrasound revealed no significant abnormalities in cardiac structures or color‐Doppler flow.

### 2.4. Electronic Ciliary Endoscopy (Capital Medical University Affiliated Beijing Children’s Hospital)

The submitted bronchial mucosal tissue shows areas with incomplete epithelial coverage. The surface exhibits sparsely arranged cilia. Most ciliary cross sections display a 9 + 2 microtubule structure with visible spokes, outer dynein arms (ODA), and partial inner dynein arms. A small proportion demonstrates 8 + 2, 9 + 1, and 7 + 2 microtubule configurations or disordered peripheral microtubule arrangements. Occasional cilia show vacuole‐like protrusions and conjoined cilia. PCD cannot be excluded.

### 2.5. Treatment Process

**FIGURE 2:**
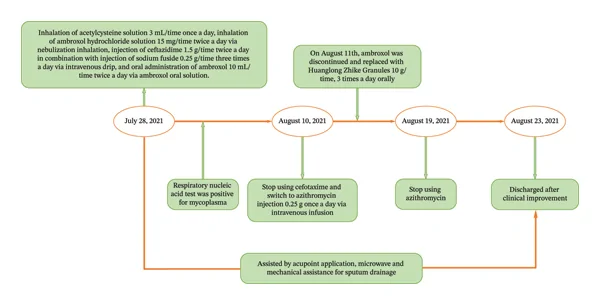
Treatment process.

Following hospitalization, symptomatic management was administered (Figure 2). The child remained afebrile with no sputum production, exhibiting only occasional single cough episodes. Clinical symptoms improved, and the patient was discharged on August 23 in stable condition.

### 2.6. Gene Variant Detection

Before admission, the child was admitted to the Beijing Children’s Hospital, Affiliated with Capital Medical University. PCD was highly suspected based on recurrent respiratory tract infection and bronchiectasis, electronic ciliary endoscopy, and other routine examinations. After obtaining the guardian’s informed consent and signing the informed consent form, the proband and his parents’ peripheral venous blood 2 mL were collected to detect the whole‐exome sequencing (WES). Sequencing analysis of disease‐related genes revealed no single‐nucleotide variants (SNVs) or copy number variations (CNVs) with strong pathogenic evidence and high correlation with the clinical phenotype. Meanwhile, additional genetic variants were identified: A heterozygous variant c.374T > C (p.Ile125Thr) was identified in Exon 4 of the CFTR gene based on the RefSeq reference transcript NM_000492.3. This variant was inherited from the father, with a global population allele frequency of 0.0128 recorded in the gnomAD database. The variant is documented in ClinVar with clinical significance annotation and is also included in the locus‐specific variant database LOVD for CFTR‐related disorders. Additionally, a heterozygous variant c.115G > T (p.Val39Leu) was detected in Exon 2 of the SFTPC gene using the standard RefSeq transcript NM_003018.3. This variant was also paternally inherited, with an allele frequency of 0.008 in the gnomAD database, and has been deposited and annotated in ClinVar and LOVD databases. Both variants were predicted to be pathogenic by bioinformatic functional prediction software (Table 1).The proband’s father and mother have no clinical phenotypes associated with PCD and present no recurrent respiratory tract infections, bronchiectasis, chronic suppurative otitis media, chronic sinusitis, or situs inversus.

**TABLE 1:** Results of whole‐exome sequencing and HGVS variant annotation.

Gene	Chromosomal location	Transcript exon	Nucleotide amino acid	Disease/phenotype (genetic mode)	Source variation
CFTR	chr7:117171053	NM_000492; exon4	c.374T > C (p.I125T)	1. {Hereditary Pancreatitis}(AD) 2. {Type 1 bronchiectasis with or without elevated sweat and chloride levels}(AD) 3. Cystic fibrosis (AR) 4. Congenital defects of the vas deferens (AR)	Father

SFTPC	Chr8:22020159	NM_003018; Exon2	c.115G > T (p.V39L)	Pulmonary surfactant dysfunction type 2 (AD)	Father

### 2.7. Isolation and Familial Genetic Analysis

All variant nomenclature in the present study strictly adheres to the HGVS standards, using NM_000492.3 for CFTR and NM_003018.3 for SFTPC as fixed reference transcripts. Population frequency data, clinical significance interpretation, and locus‐specific variation characteristics of the two variants were further validated through three authoritative public databases: gnomAD, ClinVar, and LOVD, to support genetic pathogenicity evaluation and familial segregation analysis.

With regard to the CFTR variant, it has a relatively high population allele frequency of 0.0128. Given that its associated diseases predominantly follow an autosomal recessive inheritance pattern, the heterozygous state alone is insufficient to cause disease. In addition, this variant is paternally inherited, and the carrier father presents no corresponding clinical phenotype, indicating no pathogenic segregation of this variant within the family pedigree.

As for the SFTPC variant, it has a low population allele frequency of 0.008, and its related diseases are inherited in an autosomal dominant manner, in which the heterozygous state confers potential pathogenicity. Bioinformatic functional prediction classified this variant as a likely pathogenic (D) variant, which is consistent with the pathogenic characteristics of autosomal dominant disorders. Although this variant is also inherited from the father, who lacks typical pulmonary phenotypes, this suggests incomplete penetrance. This phenomenon does not negate the potential pathogenicity of the variant but merely indicates interindividual variability in phenotypic expression.

Description of genetic variant T detection methods:

a. DNA library preparation: Genomic DNA was extracted and qualified using a Nanodrop 2000 spectrophotometer. Qualified samples were subjected to subsequent experimental procedures. A total of 1–3 μg genomic DNA was fragmented to an average size of 150 bp, followed by end repair, adapter ligation, and PCR amplification to construct the whole‐genome library.
b. Target enrichment and sequencing: The DNA library was hybridized and captured using the MyGenomics gene capture kit and 100 bp biotin‐labeled probes, which covered the coding exons as well as 50 bp flanking regions upstream and downstream of target genes. After magnetic bead washing, target DNA elution, PCR amplification, and SPRI bead purification, 150 bp paired‐end sequencing was performed on the DNBSEQ‐T7 platform.
c. Bioinformatic analysis: Raw sequencing data were stored in FASTQ format. The cutadapt software was used to trim MGI sequencing adapters and filter low‐quality reads with a length less than 80 bp. After quality control, clean reads were aligned to the human reference genome UCSC hg19 using Sentieon software. Duplicate reads were removed, and base recalibration was performed with algorithm‐driven parameters. Variant detection was conducted based on mapped reads. Sentieon software was further applied to identify SNP and InDel variants. The variant results were converted into VCF format. Finally, ANNOVAR software was used for variant annotation, and multiple public databases and bioinformatics tools were integrated to complete the pathogenicity and deleteriousness prediction of variants.
d. The whole‐genome CNV analysis: Raw sequencing data were stored in FASTQ format. The cutadapt software was used to trim MGI sequencing adapters and filter low‐quality reads with a length less than 80 bp. After quality control, clean reads were aligned to the human reference genome UCSC hg19 using Sentieon software. Duplicate reads were removed, and base recalibration was performed with algorithm‐driven parameters. Variant detection was conducted based on mapped reads. Sentieon software was further applied to identify SNP and InDel variants. The variant results were converted into VCF format. Finally, ANNOVAR software was used for variant annotation, and multiple public databases and bioinformatics tools were integrated to complete the pathogenicity and deleteriousness prediction of variants.
e. Variants selected: In this study, four steps were used to select the potential pathogenic mutations in downstream analysis: (i) Mutation reads should be more than 5, and mutation ration should be no less than 30%; (ii) the mutations should be removed, when the frequency of mutation was more than 5% in 1000 g, ESP6500, and Inhouse database; (iii) the mutations should be dropped, if they were in In Normal database (MyGenostics); (iv) the synonymous mutations should be removed, when they were not in the HGMD database. After that, the remaining mutations should be the potential pathogenic mutations for further analysis.
f. Family segregation verification: All mutations identified by DNBSEQ‐T7 sequencing were validated by Sanger sequencing. Genomic DNA was isolated from all accessible family members for subsequent Sanger sequencing.

## 3. Discussions

PCD is a disease with an abnormal ciliary structure caused by genetic variants. Cilia are “hair‐like” organelles protruding from the cell surface, which are mainly composed of axoneme, matrix, and ciliated membrane. The axoneme is the cytoskeletal structure of cilia. The cross section consists of 9 pairs of peripherally arranged doublet microtubules organized in a regular clockwise pattern, with or without a central pair of microtubules. The 9 + 0 structure cilia without a central microtubule include sensory cilia that play a role in sensory and signal transduction and nodal cilia that regulate the direction of fluid flow during embryonic development. Motile cilia with a 9 + 0 microtubule arrangement, located in the embryonic node, generate directional fluid flow through rotational movement and play a key role in regulating the establishment of left‐right body axis asymmetry during embryonic development [3].

On the other hand, motile cilia exhibit a 9 + 2 microtubule structure, consisting of 9 peripheral doublet microtubules and two central singlet microtubules. Cilia with this 9 + 2 arrangement predominantly occur in the upper and lower respiratory tracts, the female reproductive tract, and sperm flagella. Their primary functions include propelling fluid and/or mucus along epithelial surfaces (e.g., in airways) or, in the case of sperm flagella, generating the whip‐like motion required for sperm propulsion. Consequently, PCD can manifest not only as recurrent respiratory infections and bronchiectasis but also as hydrocephalus, female and male infertility/subfertility, and laterality defects (e.g., situs inversus) [1].

The incidence of PCD is about 10,000 to 20,000 [4]. The structure of cilia is complex, and there are some difficulties in diagnosis. At present, there is no gold standard in the diagnosis of PCD. Transmission electron microscopy holds diagnostic supremacy as the gold standard for PCD confirmation [5]. At the same time, gene variant detection and nasal exhaled nitric oxide (nNO) determination can improve the correct diagnosis rate of PCD. The clinical manifestations of this pediatric case include recurrent respiratory infections, bronchiectasis, and sinusitis. Transmission electron microscopy of bronchial mucosal tissue revealed focal areas with incomplete epithelial coverage. The cilia on the surface showed a sparse arrangement, with some cross‐sectional views demonstrating abnormal microtubule configurations (8 + 2, 9 + 1, and 7 + 2 patterns) and disorganized peripheral microtubules. Researchers identified vacuolar protrusions in a subset of cilia and detected conjoined ciliary structures through microscopic analysis. Clinicians could not exclude PCD. The diagnostic team integrated abnormal lung function parameters with characteristic clinical findings to establish a definitive PCD diagnosis.

The ultrastructure of cilia under an electron microscope is the most commonly used method for diagnosing PCD. Common ciliary structural defects include ODA, ODA + inner dynein arms, and inner dynein arms + peripheral microtubule pair defects. In contrast, central microtubule and microtubule connection protein defects are relatively rare. The most common ciliary ultrastructural abnormality is the absence of ODA. Published studies document that 40% (20/50) of analyzed ciliary ultrastructural defect cases manifest ODA deficiency, with this finding being reproducibly demonstrated across peer‐reviewed case series [6]. Structural protein‐related genes (*DNAH5*, *DNAI1*, *DNAI2*, and *NME8*) [7–9], dynein arm docking complex‐related genes (*CCDC114*, *CCDC151*, and *ARMC4*, *TTC25*) [10, 11], and the *CCDC103* gene [12] can all manifest as ODA defects. Literature reports indicate that related genes such as *DNAAF1*, *DNAAF2*, *DNAAF3*, *DYX1C1*, and *HEATR2* [13, 14] may also present with ODA absence, but this condition is often accompanied by inner dynein arm deficiency.

So far, more than 50 genes characterized by motor ciliary dysfunction are involved, from 33 PCD‐related gene variants known in 2018 to pathogenic variants of 50 genes known to cause PCD and its associated active cilia [5]. Research teams worldwide continually identify novel PCD‐associated gene variants, with these molecular discoveries enabling refined diagnostic protocols for PCD. The genetic mode of PCD is usually autosomal recessive inheritance, but there are also some examples of X‐linked and dominant inheritance. For these genes related to PCD variants, different gene variants may have different phenotypes [2].

Standardized reporting of genetic variants relies on unified HGVS nomenclature and fixed RefSeq reference transcripts. The transcripts NM_000492.3 (CFTR) and NM_003018.3 (SFTPC) adopted in this study are widely recognized standard reference sequences for genetic variant annotation [15]^.^ At present, the interpretation of genetic variants in PCD and pediatric respiratory genetic diseases is highly dependent on mainstream public variation resources: gnomAD provides large‐scale population allele frequency data, ClinVar offers authoritative clinical significance annotation, and LOVD supports locus‐specific variant collation and genotype–phenotype correlation analysis [16–18].

Regarding the two gene variants in the gene variant detection of this case, the *CFTR* gene variant has been reported in the literature database as being related to cystic fibrosis. Zhu Xinghai and others reported that a case of cystic fibrosis caused by *CFTR* gene variants was mainly manifested as repeated coughing, wheezing, and repeated pulmonary infections [19]. Previous related literature reported that the *SFTPC* gene variant can cause interstitial pulmonary disease. Luis Rodriguez and others showed that *SFRPC* gene variants can lead to pulmonary fibrosis in mice [20]. This pediatric patient presented with recurrent respiratory infections accompanied by bronchiectasis and sinusitis. Abdominal and cardiac ultrasound revealed no situs inversus. Diagnosticians confirmed PCD using transmission electron microscopy, identifying hallmark ciliary ultrastructural defects characteristic of the disorder. Genetic variant detection identified gene variants in the *CFTR* and *SFTPC* genes, suggesting a potential mechanistic link between these genetic alterations and PCD pathogenesis. Further validation via targeted animal studies is warranted to elucidate this observed genotype‐phenotype correlation.

In addition, the nNO of children diagnosed with PCD was significantly decreased [21]. As a noninvasive, fast, and inexpensive test, nNO testing is recommended by the European Respiratory Association and the American Thoracic Society as a front‐line screening test for PCD [22]. The diagnostic threshold of 77 nL/min recommended by Leigh is widely used worldwide [23]. The decrease of nNO in children with PCD may be related to the disturbance of NO diffusion caused by abnormal mucus clearance in the respiratory tract, the abnormal expression or decreased activity of NO synthase in the respiratory tract, or the blockage of the sinus orifice caused by repeated infection or mucus clearance disturbance [24].

## 4. Conclusions

Current evidence suggests that the *CFTR* and *SFTPC* gene variants may be associated with PCD in children. Mechanistic studies are ongoing to elucidate their synergistic effects on the mucociliary clearance pathway. The main clinical manifestations include recurrent respiratory infections, bronchiectasis, sinusitis, etc. PCD is a rare hereditary disease. Clinicians risk overlooking or misclassifying cases when specific visceral translocation fails to manifest characteristic clinical indicators, particularly during early diagnostic evaluations. Therefore, it is essential to enhance clinicians’ awareness of PCD. Pediatric pulmonologists must progressively refine nasal exhaled nitric oxide measurement protocols and ciliary ultrastructural analysis via transmission electron microscopy when managing children exhibiting recurrent respiratory infections, bronchodilator dependence, and chronic sinusitis. Geneticists must prioritize enhancing genomic variant diagnostics for PCD to bolster diagnostic accuracy and expand the disease’s mutation spectrum, implementing protocol optimizations within targeted timeframes when clinically indicated.

## Author Contributions

Dan Shao and Xinhui Yuan contributed to the conception and design of the study. Dan Shao drafted and edited the manuscript. Dan Shao, Yumei Li, Xinhui Yuan, Jizu Ling, and Yigu Gong interpreted and analyzed the data.

## Funding

No funding was used in this study.

## Disclosure

All authors reviewed the manuscript and approved the final version.

## Ethics Statement

This study has been reviewed and approved by the Ethics Committee of the First Hospital of Lanzhou University (approval no.: LDYYLL‐2025‐161) and strictly adheres to the principles outlined in the Declaration of Helsinki.

## Consent

Written informed consent was obtained from the patient’s legal guardian for publication of this case report and any accompanying images. A copy of the written consent is available for review by the Editor‐in‐Chief of this journal.

## Conflicts of Interest

The authors declare no conflicts of interest.

## Data Availability

Research data are not shared.

## References

[1] Raidt J. , Loges N. T. , Olbrich H. , Wallmeier J. , Pennekamp P. , and Omran H. , Primary Ciliary Dyskinesia, Presse Medicale. (2023) 52, no. 3, 10.1016/j.lpm.2023.104171. 37516247

[2] Brennan S. K. , Ferkol T. W. , and Davis S. D. , Emerging Genotype-Phenotype Relationships in Primary Ciliary Dyskinesia, International Journal of Molecular Sciences. (2021) 22, no. 15, 10.3390/ijms22158272. PMC8348038 34361034

[3] Basu B. and Brueckner M. , Cilia Multifunctional Organelles at the Center of Vertebrate left-right Asymmetry, Current Topics in Developmental Biology. (2008) 85, 151–174, 10.1016/S0070-2153(08)00806-5. 19147005

[4] Horani A. and Ferkol T. W. , Advances in the Genetics of Primary Ciliary Dyskinesia: Clinical Implications, Chest. (2018) 154, no. 3, 645–652, 10.1016/j.chest.2018.05.007. 29800551 PMC6130327

[5] Collaborative Group on Difficult and Rare Diseases of the Respiratory Science Branch of the Chinese Medical Association , National Clinical Research Center for Respiratory Diseases , and Editorial Committee of the Chinese Journal of Practical Pediatric Clinical Medicine , Expert Consensus on Diagnosis and Treatment of Primary Ciliary Dyskinesia in Children, Chinese Journal of Practical Pediatrics. (2018) 33, 94–99.

[6] Pifferi M. , Bush A. , Mulé G. et al., Longitudinal Lung Volume Changes by Ultrastructure and Genotype in Primary Ciliary Dyskinesia, Annals of the American Thoracic Society. (2021) 18, no. 6, 963–970, 10.1513/annalsats.202007-816oc. 33760720

[7] Vanaken G. J. , Bassinet L. , Boon M. et al., Infertility in an Adult Cohort With Primary Ciliary Dyskinesia: Phenotype-Gene Association, European Respiratory Journal. (2017) 50, no. 5, 10.1183/13993003.00314-2017. 29122913

[8] Al-Mutairi D. A. , Alsabah B. H. , Alkhaledi B. A. , Pennekamp P. , and Omran H. , Identification of a Novel Founder Variant in DNAI2 Cause Primary Ciliary Dyskinesia in Five Consanguineous Families Derived From a Single Tribe Descendant of Arabian Peninsula, Frontiers in Genetics. (2022) 13, 10.3389/fgene.2022.1017280. PMC9596166 36303540

[9] Duriez B. , Duquesnoy P. , Escudier E. et al., A Common Variant in Combination with a Nonsense Mutation in a Member of the Thioredoxin Family Causes Primary Ciliary Dyskinesia, Proceedings of the National Academy of Sciences of the United States of America. (2007) 104, no. 9, 3336–3341, 10.1073/pnas.0611405104. 17360648 PMC1805560

[10] Hjeij R. , Onoufriadis A. , Watson C. M. et al., CCDC151 Mutations Cause Primary Ciliary Dyskinesia by Disruption of the Outer Dynein Arm Docking Complex Formation, The American Journal of Human Genetics. (2014) 95, no. 3, 257–274, 10.1016/j.ajhg.2014.08.005. 25192045 PMC4157146

[11] Wallmeier J. , Shiratori H. , Dougherty G. W. et al., TTC25 Deficiency Results in Defects of the Outer Dynein Arm Docking Machinery and Primary Ciliary Dyskinesia with Left-Right Body Asymmetry Randomization, The American Journal of Human Genetics. (2016) 99, no. 2, 460–469, 10.1016/j.ajhg.2016.06.014. 27486780 PMC4974089

[12] Pereira R. , Oliveira M. E. , Santos R. et al., Characterization of CCDC103 Expression Profiles: Further Insights in Primary Ciliary Dyskinesia and in Human Reproduction, Journal of Assisted Reproduction and Genetics. (2019) 36, no. 8, 1683–1700, 10.1007/s10815-019-01509-7. 31273583 PMC6708006

[13] Aprea I. , Raidt J. , Höben I. M. et al., Defects in the Cytoplasmic Assembly of Axonemal Dynein Arms Cause Morphological Abnormalities and Dysmotility in Sperm Cells Leading to Male Infertility, PLoS Genetics. (2021) 17, no. 2, 10.1371/journal.pgen.1009306. PMC7909641 33635866

[14] Horani A. , Gupta D. K. , Xu J. et al., The Effect of Dnaaf5 Gene Dosage on Primary Ciliary Dyskinesia Phenotypes, JCI Insight. (2023) 8, no. 11, 10.1172/jci.insight.168836. PMC10393236 37104040

[15] Pruitt K. D. , Tatusova T. , and Maglott D. R. , NCBI Reference Sequence (RefSeq): A Curated Non-Redundant Sequence Database of Genomes, Transcripts and Proteins, Nucleic Acids Research. (2005) 33, D501–D504, 10.1093/nar/gki025. 15608248 PMC539979

[16] Landrum M. J. , Lee J. M. , Benson M. et al., ClinVar: Improving Access to Variant Interpretations and Supporting Evidence, Nucleic Acids Research. (2018) 46, no. D1, D1062–D1067, 10.1093/nar/gkx1153. 29165669 PMC5753237

[17] Karczewski K. J. , Francioli L. C. , Tiao G. et al., The Mutational Constraint Spectrum Quantified from Variation in 141,456 Humans, Nature. (2020) 581, no. 7809, 434–443, 10.1038/s41586-020-2308-7. 32461654 PMC7334197

[18] Fokkema I. F. A. C. , Taschner P. E. M. , Schaap M. et al., LOVD: Easy Creation of Locus-specific Mutation Databases and Variation Curation, Human Mutation. (2021) 42, no. 1, 5–13.

[19] Zhu X. H. and Yang Y. , One Case of Cystic Fibrosis, Journal of Clinical Pulmonary Medicine. (2025) 30, 157–159.

[20] Rodriguez L. , Tomer Y. , Carson P. et al., Chronic Expression of a Clinical SFTPC Mutation Causes Murine Lung Fibrosis With Idiopathic Pulmonary Fibrosis Features, American Journal of Respiratory Cell and Molecular Biology. (2023) 68, no. 4, 358–365, 10.1165/rcmb.2022-0203ma. 36473455 PMC10112421

[21] Collins S. A. , Gove K. , Walker W. , and Lucas J. S. , Nasal Nitric Oxide Screening for Primary Ciliary Dyskinesia: Systematic Review and Meta-Analysis, European Respiratory Journal. (2014) 44, no. 6, 1589–1599, 10.1183/09031936.00088614. 25323224

[22] Shapiro A. J. , Davis S. D. , Polineni D. et al., Diagnosis of Primary Ciliary Dyskinesia. An Official American Thoracic Society Clinical Practice Guideline, American Journal of Respiratory and Critical Care Medicine. (2018) 197, no. 12, e24–e39, 10.1164/rccm.201805-0819st. 29905515 PMC6006411

[23] Leigh M. W. , Hazucha M. J. , Chawla K. K. et al., Standardizing Nasal Nitric Oxide Measurement as a Test for Primary Ciliary Dyskinesia, Annals of the American Thoracic Society. (2013) 10, no. 6, 574–581, 10.1513/annalsats.201305-110oc. 24024753 PMC3960971

[24] Walker W. T. , Jackson C. L. , Lackie P. M. , Hogg C. , and Lucas J. S. , Nitric Oxide in Primary Ciliary Dyskinesia, European Respiratory Journal. (2012) 40, no. 4, 1024–1032, 10.1183/09031936.00176111. 22408195

